# Assessment of Mineralogical Characteristics of Clays and the Effect of Waste Materials on Their Index Properties for the Production of Bricks

**DOI:** 10.3390/ma15248908

**Published:** 2022-12-13

**Authors:** Aamar Danish, Ermedin Totiç, Muhammed Bayram, Mücahit Sütçü, Osman Gencel, Ertuğrul Erdoğmuş, Togay Ozbakkaloglu

**Affiliations:** 1 Ingram School of Engineering, Texas State University, San Marcos, TX 78666, USA; 2 Civil Engineering Department, Faculty of Engineering, Architecture and Design, Bartin University, Bartin 74100, Turkey; 3 Materials Science and Engineering Department, Faculty of Engineering and Architecture, Izmir Kâtip Celebi University, Izmir 35620, Turkey; 4 Environmental Engineering Department, Faculty of Engineering, Architecture and Design, Bartin University, Bartin 74100, Turkey

**Keywords:** clay, brick production, index properties, mineralogy, waste materials

## Abstract

Significant research investigations on the characteristics of unexplored clay deposits are being conducted in light of the growing need for clay in the ceramic industry and the variable chemistry of clays. Parallel to this, the generation of waste materials like fly ash, ferrochrome slag, and silica fume is also increasing, responsible for environmental degradation. This paper aims to study the mineralogical properties of pure clays (one specimen from Siberia and five specimens from different locations in Turkey), and the effect of mentioned waste materials on the index properties of clays obtained. This study is divided into two phases, wherein in the first phase, the pure clay specimens are analyzed against mineralogical properties (i.e., chemical composition, thermal analysis, and particle size distribution). While in the second phase, index properties of pure clay specimens and clay specimens modified with 0–50% fly ash, ferrochrome slag, and silica fume are analyzed. The results reveal that the clay specimens from Turkey (USCS classification: CL) are fit for the ceramic industry and bricks production, and incorporation of waste materials can further improve their index properties. It is also observed that incorporation of 10–30% fly ash and ferrochrome slag have higher efficiency in reducing the plasticity index of clays studied as compared to the addition of silica fume.

## 1. Introduction

Clay is a ubiquitous natural mineral that has been utilized in a wide range of applications in different industries since the ancient era [1]. The study of clay minerals independently, as soil components, and fine-grained rocks is essential to understand various biological [2,3], environmental [4], and sedimentary processes [5] worldwide. Apart from the geological viewpoint, clays are important segments of our lives due to their enormous usage and peculiar physiochemical characteristics. The manmade utilization of clay dates to prehistoric times where it was used for making pottery and writing tablets. Nowadays, the usage of clay is so diverse that it covers sectors such as construction materials [6], geopolymers [7,8,9], ceramics [10], civil engineering [11], cosmetics [12], chemicals [13], environment [14], or health [15], among many others.

Huge deposits of clay on the surface of the earth are generally defined as material consisting of hydrous phyllosilicates having a size less than 2 μm [16]; however, clay is a material with variable chemistry in which investigators from various fields still disagree with the definition of clay. For example, sedimentologists believe that clay can be categorized by particles having a size (in terms of equivalent spherical diameter) less than 4 μm; on the other hand, chemists and geologists believe that clays should have a particle size less than or equal to 1 μm and 2 μm, respectively [17]. It noteworthy that the mentioned conflicting viewpoints can also be found in the most widely used procedures for determining clay content, but conventional sedimentation tests overestimate the percentage of clay content and sophisticated methods (like laser-diffraction) underestimate the clay percentage [18]. This is because the utilization of clay as a raw material (e.g., construction and ceramic industry) in environmental protection (e.g., absorbent materials and medium for crops in agricultural industry) or as a study object in geotechnical engineering (e.g., mechanics of soil) is characterized by a high level of complexity owing to the interaction between different natures of gas, liquid, and solid phases leading to intricate behavior, which is difficult to understand. For example, clay minerals like kaolins, palygorskite, sepiolite, and smectites have distinct compositions and structure despite the fact that they have similar basic building blocks (i.e., octahedral and tetrahedral sheets). However, the composition and structure of these octahedral and tetrahedral sheets are responsible for minor or major changes in the physiochemical characteristics of the mentioned clay minerals [19]. This complexity increases for the clay deposits with no or negligible data.

Brick belongs to a broad family of construction and building materials and is primarily used to construct inner and outer walls of infrastructures [20]. Bricks can also be used for constructing load-carrying walls because some countries (such as Turkey and Ukraine) allow the construction of three-story buildings with bricks [21]. Bricks are recommended for construction activities owing to the ease of production, easy accessibility of raw materials, excellent engineering properties such as compressive strength, fire resistance, durability, low maintenance cost, and serviceability [22]. Additionally, bricks also provide moderate insulating property by preventing heat transfer in hot weather [23].

In countries like Turkey and Spain, one of the most important industries is the ceramic industry with bricks as one of the most widely produced product. The production of traditional clay-based bricks commonly uses shale and clay mixtures as the raw materials, which demand processes like shaping, drying, and high-temperature firing. Due to increasing industrialization, urbanization, and economic growth, the requirement for building materials is increasing rapidly. For example, the global yearly production of bricks is currently reported to be approximately 1400 billion units, and it is predicted to increase continuously for the upcoming years [24]. The production of bricks is even greater in developing countries, i.e., more than 87% of globally used bricks are produced in Asia and around 20% of those are made in South Asian countries (such as Bangladesh, India, Nepal, and Pakistan) [25]. The production of bricks in mentioned countries is mainly conducted via small-scale conventional kilns to burn fossil fuels without any environmental protection protocols [26]. In the production of bricks, clay is the most abundantly used raw material, which has caused immense exploitation of natural clay deposits. In addition, brick production requires cultivatable clay which is a threat to the future of agriculture in different countries like China and India [27]. Although natural clay reserves (explored or unexplored) are available in many countries, a continual increase in brick production can cause shortage of natural clay resources [28]. Therefore, the brick industry is looking for an optimal solution to produce sustainable and economical bricks (such as 3D printed clay bricks [29,30], industrial waste modified clay bricks [31,32,33], etc.) with possible enhancement in mechanical and thermal performance.

Parallel to this, the world is facing severe environmental challenges caused by the accumulation of unmanaged wastes [28]. This problem is more pronounced in developing countries (such as India and Pakistan) where open landfill sites are common scenes owing to the lack of skilled labor and low budget for sustainable waste disposal. The accumulation of waste is detrimental to human health and the environment unless stored, managed, or disposed of in a sustainable manner [34]. Even though regulations related to the disposal of hazardous effluents and wastes are available, solid wastes (such as fly ash (FA), silica fume (SF), and ferrochrome slag (FS)) are commonly disposed of indiscriminately into the environment, which may pose detrimental health risks. It is important to note that though FA has a great importance to the cement industry, FA-modified cement and related products are not fully commercialized worldwide.

FA is the most abundantly produced industrial waste, which contains toxic and complex anthropogenic constituents making it difficult to manage [35]. As per an estimate, about 800 million tons of FA is globally generated every year, but only 200 million tons is reused in different applications while the remaining 600 million tons of FA is dumped on surface impoundments and large open sites [36]. Similarly, 6.5–9.5 million tons of ferrochrome is being produced worldwide annually, with an increasing rate of 3% per year [37]. This is increasing ferrochrome waste, which is mostly landfilled leading to environmental pollution without any appreciable attention towards control and mitigation. The most plentifully produced wastes of the ferroalloy industry are FS and ferrochrome ash (FEA). The production of 1 ton of ferrochrome product generates 1–1.2 tons of FS and 0.02–0.03 tons of FEA [37]. SF is another type of industrial waste produced during the smelting process in the silicon industry [38]. The annual worldwide production of SF is reported to be 1 million tons with a major contribution from the U.S. (130,000 tons) and Norway (120,000 tons) [39].

The index properties of clay are important because they influence the preparation process, properties, and performance clay-based products. For example, there is a risk of high expansion or compression in clays with a high liquid limit (LL) leading to reduced life of clay-based products [40]. Similarly, high and low plasticity index (PI) of clays leads to reduced shear-strength and appreciable change in consistency, which limits the usability of such clays for the brick production [41]. Previously published studies have evaluated the influence of industrial wastes on the index properties of clays [40,42,43,44,45,46,47,48,49,50,51]. For instance, Phanikumar and Nagaraju [52] studied the effect of index properties of FA- and rice husk ash (RHA)-modified expansive clay. The study revealed that incorporation of FA reduced the LL and PI of clay by 48%, and 81%, respectively. While the addition of RHA decreased the LL and PI of clay by 64% and 93%, respectively. Contrary to this, the incorporation of FA and RHA increased the plastic limit (PL) of clay by 16% and 21%, respectively. Fattah et al. [53] investigated the impact of RHA on the characteristics of clayey soils. They observed that the addition of 9% RHA decreased the LL and PI of clayey soil by 11–18% and 32–80%, respectively. Kalkan [54] studied the index properties of SF-modified clayey soils. He concluded that 10–30% SF reduced the LL and PI of clayey soils by 12.3% and 30%, respectively, and caused negligible change in PL.

In view of the increasing demand for clay mainly for brick production, and the increasing generation of industrial wastes, research should be conducted on the characterization of unexplored deposits of clay modified with industrial wastes. Through this experimental study, we are proposing a sustainable alternate for the safe disposal of the three most abundantly produced waste (FA, SF, and FS) by integrating them partially in six different rarely explored deposits of clays in Siberia and Turkey. The main goal is to investigate the mineralogical and index properties of clays with the mentioned industrial wastes for the production of ceramic products (especially bricks).

## 2. Materials and methods

### 2.1. Materials

#### 2.1.1. Clay

**Figure 1:**
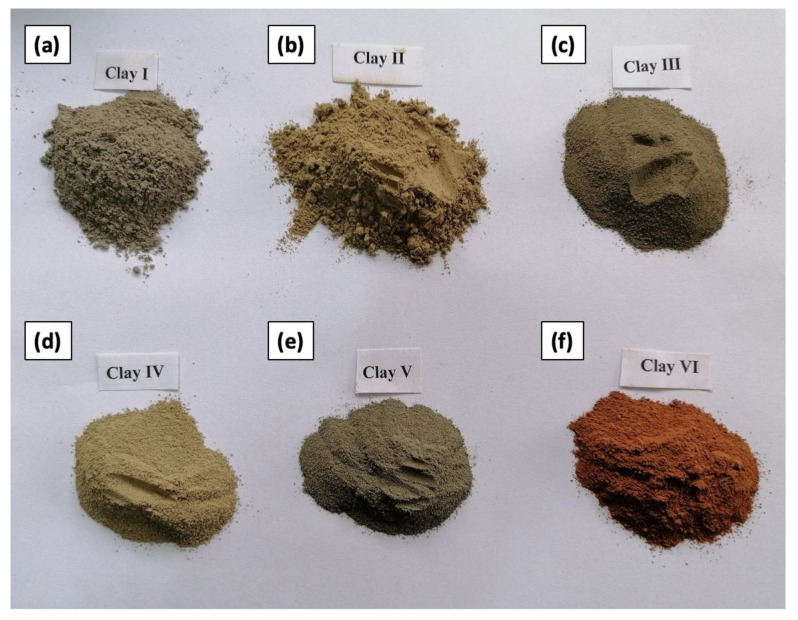
Digital image of (**a**) Clay I, (**b**) Clay II, (**c**) Clay III, (**d**) Clay IV, (**e**) Clay V, and (**f**) Clay VI.

Five clay samples were acquired from different locations in Turkey, i.e., Bartin University, Üçyildiz, Işıklar, Boyabat, and the sixth sample from Serbia. The clay specimens were collected from 1.5 m below the ground level. The disturbed clay specimens collected from the mentioned sites were kept in airtight plastic bags while transported to the laboratory for experimentation. Airtight bags were selected to avoid the interaction of clay specimens with the environment and to reduce the risk of importing impurities. Table 1 shows the physical properties of clay specimens. The digital images of clays used in this study are shown in Figure 1.

#### 2.1.2. Industrial Waste

**Figure 2:**
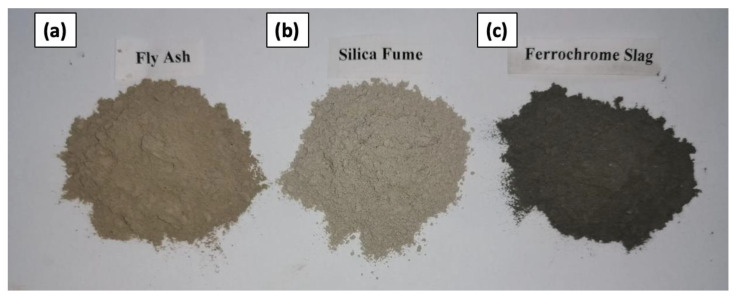
Digital image of (**a**) FA, (**b**) SF, and (**c**) FS.

**Figure 3:**
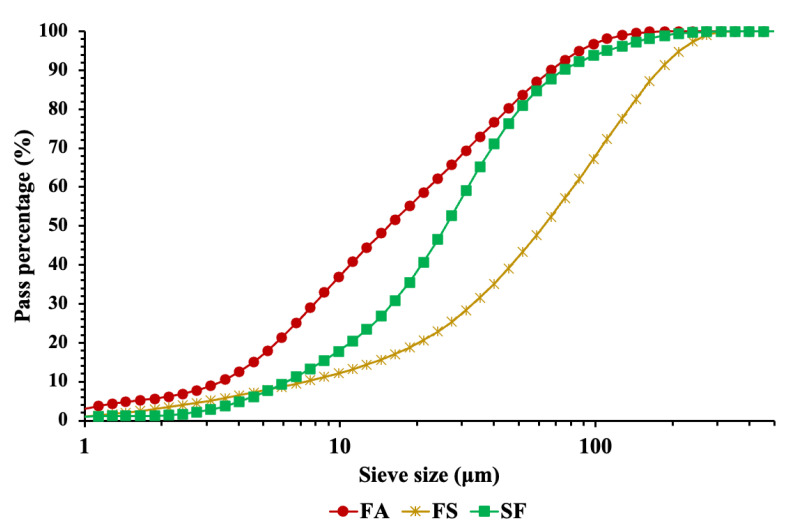
Particle size distribution of FA, FS, and SF.

Industrial wastes such as FA, FS, and SF were acquired from the local brick, ferrochrome, and silicon industry. The physical properties of FA, FS, and SF are listed in Table 2. Figure 2a–c represents the digital image of FA, SF, and FS, respectively. The particle size distribution of industrial wastes is shown in Figure 3.

**Figure 4:**
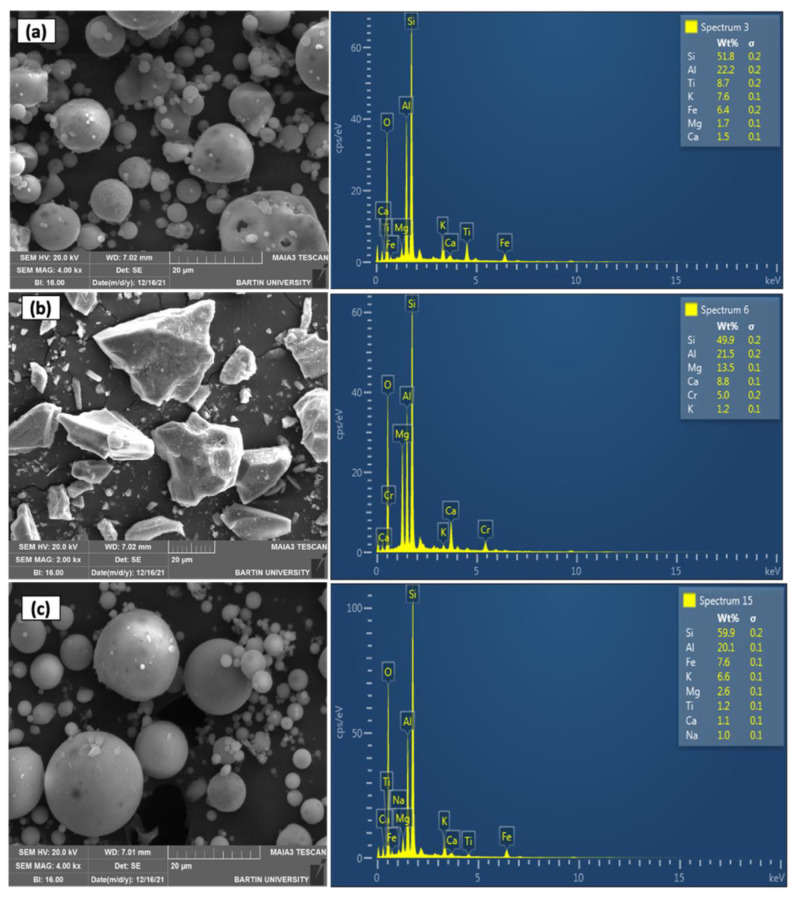
SEM and EDX analysis of (**a**) FA, (**b**) FS, and (**c**) SF.

The scanning electron microscope (SEM) and Energy Dispersive X-ray (EDX) analysis of FA, FS, and SF were conducted to assess their topography and elemental composition, as shown in Figure 4a–c, respectively. The SEM images (taken at the magnification of 20 µm) reveals that the particle shape of FA is spherical with pores of varying diameters and several small size particles adhere to the large size particles (see Figure 4a). The EDX image shows that FA is composed of high quantity silicon (Si) (51.8 wt%) and aluminum (Al) (22.2 wt%), moderate quantity of titanium (Ti) (8.7 wt%), potassium (K) (7.6 wt%), iron (Fe) (6.4 wt%) and minor fractions of magnesium (Mg) (1.7 wt%) and calcium (Ca) (1.5 wt%). On the other hand, the topography of FS particles is rough with an angular particle shape, as shown in Figure 4b. Like FA, FS also contains a high amount of Si (49.9 wt%) and Al (21.5 wt%) but contains an adequate amount of Mg (13.5 wt%), Ca (8.8%), Chromium (Cr) (5.0 wt%) and a minimal amount of K (1.2 wt%). The particle shape and topography of SF is way more spherical and smoother than FA, respectively (see Figure 4c). The elemental composition of SF contains Si, Al, Fe, K, Mg, Ti, Ca, and sodium (Na) in an amount of 59.9 wt%, 20.1 wt%, 7.6 wt%, 6.6 wt%, 2.6 wt%, 1.2 wt%, 1.1 wt%, 1 wt%, respectively.

**Figure 5:**
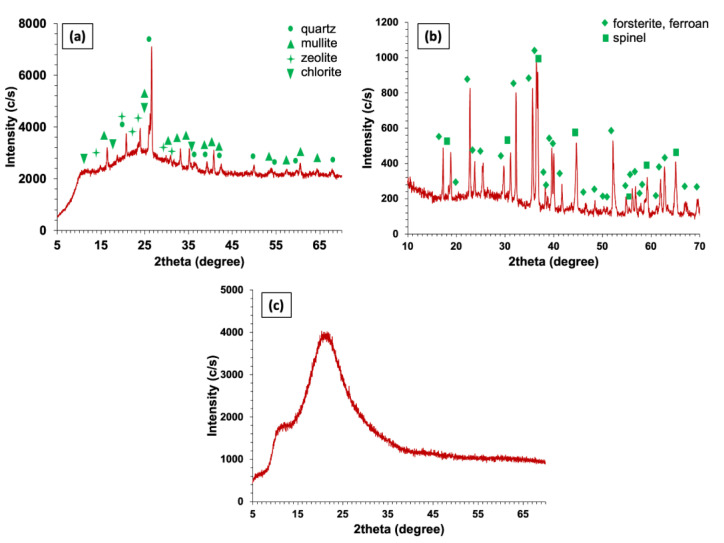
XRD patterns of (**a**) FA, (**b**) FS, and (**c**) SF.

Figure 5a–c represents the XRD patterns of FA, FS, and SF, respectively. Figure 5a shows that FA is mainly composed of quartz, mullite, zeolite, and chlorite. Figure 5b shows that FS constitutes of forsterite (ferroan), whereas the XRD pattern of SF shows that it has an amorphous structure with no peaks, as shown in Figure 5c. The database of the International Centre for Diffraction Data (ICDD) (see Table A1 in Appendix A) was used to determine the XRD peaks.

### 2.2. Testing Methods

#### 2.2.1. X-ray Fluorescence (XRF)

The chemical composition of clays and waste materials was investigated via Spectro, Xepos II. The grain size of every specimen (<0.5 g) less than 100 μm was used for this analysis, which was mixed with an organic binder (3 drops) and hydraulically pressed. Finally, the specimens were loaded in the sample chamber of the XRF spectrometer with a maximum current and voltage of 30 kV and 1 mA for 10 min.

#### 2.2.2. X-ray Diffraction (XRD)

For XRD test, clays and waste materials were dried in an oven at 105 °C for 1 h and cooled in different desiccators. Each specimen with particle size less than 2 μm cross-section was pressed in a sample holder to get a flat smooth surface. Some portion of the prepared specimen was placed on a glass slide to obtain strong basal reflections of minerals in clays and waste materials. XRD analysis of clay and waste materials was conducted using Rigaku SmartLab device Cu Kα (λ = 0.154 nm) radiating at 2θ with intervals between 5° and 70° and scanning speed of 0.04°/s.

#### 2.2.3. Thermogravimetric Analysis (TGA)

To assess the mass of clay raw materials and waste materials as a function of temperature, thermogravimetric analysis (TGA) was performed. For this study, TGA was performed using Perkin-Elmer STA8000 equipment on specimens heated in a nitrogen atmosphere at 950 °C with purge rate of 20 mL/min and temperature increment of 10 °C/min.

#### 2.2.4. Particle Size Distribution (PSD)

Malvern Mastersizer 2000 manufactured by Malvern Instruments was used as a laser particle analyzer for clays and waste materials. This device works on the basis of Mie Theory to obtain particle size distribution by converting light scattering data. Along with Malvern Mastersizer 2000, HydroG was used as a specimen dispersion unit. Specimens of clay and waste materials were processed using Elix filtered water in known amounts and were run at a speed between 900–2000 rpm. In order to reduce the residual error, the absorption and refractive index of Malvern Mastersizer 2000 was set to 0.008 and 1.544, respectively.

#### 2.2.5. Atterberg Limits

Atterberg limits such as LL, PL, and shrinkage limit (SL) were conducted according to the specifications mentioned in ASTM 4318 [55]. The PI for each specimen was calculated by subtracting PL from LL as per the ASTM 4318 [55]. To maintain the accuracy of data, the mentioned tests were conducted twice under similar test procedures and controlled laboratory conditions.

### 2.3. Specimen Preparation

All clays and wastes materials were processed by drying in an oven at the temperature of 105 ± 5 °C for 24 h to remove moisture. The dried wastes were then grounded repeatedly using a plastic hammer to remove any clusters. To prepare the mixture of clays with materials, the required quantity of clays and waste materials were measured and mixed in a dry state. The quantities of waste materials were selected between 0–50% with an increment of 10%. Manual mixing was performed, and appropriate care was taken to prepare homogenous mixtures at each mixing stage.

## 3. Results and Discussion

### 3.1. Mineralogical Properties

#### 3.1.1. X-ray Fluorescence

**Figure 6:**
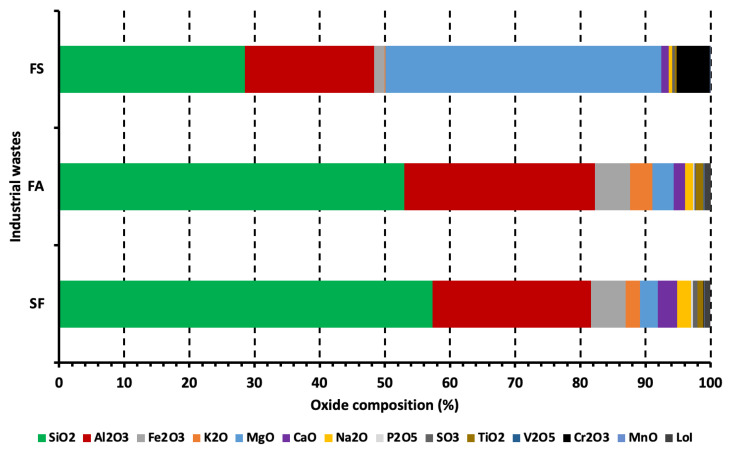
Chemical composition of industrial wastes.

The chemical composition of industrial waste materials assessed in this work is shown in Figure 6. The chemical composition of FA and SF is approximately the same while the chemical composition of FS is different (see Figure 6). For example, SF and FA have an abundant quantity of silicon dioxide (SiO_2_) and aluminum oxide (Al_2_O_3_), whereas FS has a greater amount of magnesium oxide (MgO), SiO_2_, and Al_2_O_3_. It is important to note that the presence of calcium oxide (CaO), especially in SF, may react with Si/Al in clay in the presence of moisture to produce cementitious composites leading to the enhancement in strength properties and decrement in the swell potential of clay [56]. Similarly, the difference in oxide composition and mineral phases between clays and waste materials may cause different firing parameters, properties, and performance between waste material-clay bricks and pure clay bricks.

**Figure 7:**
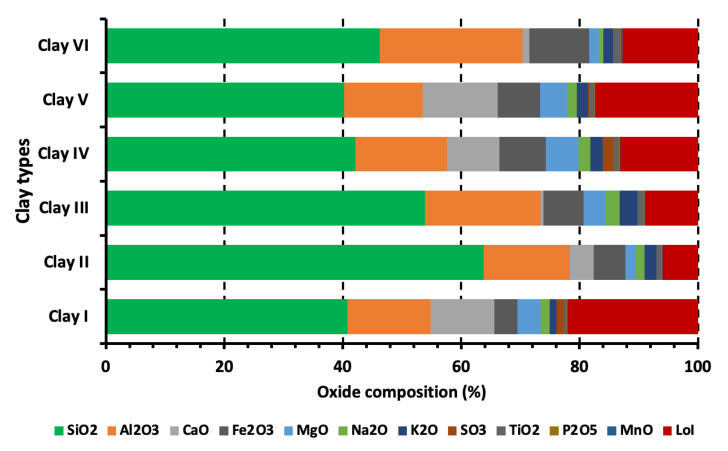
Chemical composition of clays.

The chemical composition of clays studied in this research work is shown in Figure 7, where it can be observed that SiO_2_ and Al_2_O_3_ are the most abundant oxides in all six clays. Clay II has the highest amount of SiO_2_ while Clay V has the minimum quantity of SiO_2_, which is related to the presence of quartz in the clay specimen. The lowest and highest quantity of Al_2_O_3_ can be observed in Clay V (13.26%) and Clay VI (24.14%), respectively. It noteworthy that Clay VI is the only clay type with Al_2_O_3_ greater than 20%, which can be ascribed to the presence of Kaolinite. However, Clay I shows abnormal characteristic, wherein it has a lower amount of Al_2_O_3_ as compared to other clay types but still has kaolinite in the XRD patterns (see Section 3.1.2). Apart from SiO_2_ and Al_2_O_3_, an appreciable amount of CaO, iron (III) oxide (Fe_2_O_3_), MgO, sodium oxide (Na_2_O), and potassium oxide (K_2_O) are also present, with traces of sulfur trioxide (SO_3_), titanium dioxide (TiO_2_), phosphorous pentoxide (P_2_O_5_), and manganese oxide (MnO).

All clay types have siliceous nature that can be attributed to SiO_2_ being the main component with minor quantities of Al_2_O_3_ and Fe_2_O_3_. The SiO_2_/Al_2_O_3_ ratios for the investigated clays (except Clay VI) are higher than the values observed in montmorillonite (SiO_2_/Al_2_O_3_: 2.36) and pure kaolinite (SiO_2_/Al_2_O_3_: 1.18) but close to ones observed in the clays from South Africa [57] and Cambodia [58].

The presence of an appreciable amount of CaO, Fe_2_O_3_, MgO, Na_2_O, and K_2_O in all clay types is advantageous to produce fired-clay bricks because the mentioned oxides act as fluxes, which decrease the temperature needed to produce glassy material on the surface of the brick. In other words, the presence of the mentioned oxides decreases the melting point of clay [59]. Clay VI may show minimum fluxing behavior because it has a minimum quantity of alkaline earth oxides (CaO and MgO) and alkali oxides (Na_2_O and K_2_O), which is the consequence of being a kaolinitic clay [60]. Clay III has an appreciable quantity of Al_2_O_3_ and an amount of CaO less than 1%, this can aid in sintering during the firing process, which supports its utilization in brick, stoneware, and tiles production [61]. Moreover, the lowest quantity of CaO in Clay III as compared to other clay types indicate that it has no or negligible quantity of carbonate inclusions. On the other hand, though other clay types (except Clay III) have both Al_2_O_3_ (>13.26%) and CaO (>4.0%) in a higher amount, it may be detrimental for sintering during the firing process [10].

Apart from the fluxing function of Fe_2_O_3_, it is also a major colorant agent, and responsible for providing red color to clay products after firing [62,63]. Based on Fe_2_O_3_ quantity in clays studied, ceramic wares prepared from Clay I may have the lighter color and Clay VI may have brighter color. This is a vital characteristic because it renders potential use of Clay I in the production of products having cream tonality, particularly in rustic floor and roofing tiles. The lower proportion of Fe_2_O_3_ (e.g., Clay I) either due to isomorphic substitution in clay mineral structures, or a low amount of iron oxide, which was undetectable [64]. While a higher proportion of Fe_2_O_3_ (e.g., Clay II–VI) might be because of the alteration and weathering of any nearby basalt rocks [58]. On the other hand, few researchers have reported that the quantity of Fe_2_O_3_ should not be considered as the only parameter for coloration of clay products because other constituents of clay (such as CaO, MgO, and MnO), the quantity of Al_2_O_3_ relative to other oxides, the atmosphere of the furnace play an essential role as well [65,66].

During the conduction of loss on ignition (LoI) test on clay types, carbon dioxide might have been released, which could make them less acidic. As per Figure 7, Clay II/III, Clay IV/VI, and Clay I/V have low, moderate, and high LoI values, respectively, ranging between 6% (Clay II) to 22% (Clay I). The LoI value of clay depends on the oxidation of organic matter, dehydroxylation of the clay minerals, and decomposition of carbonates, hydroxides, sulfides, etc. [67]. The weight loss of clays studied will be discussed in detail in thermogravimetric analysis (see Section 3.1.3).

#### 3.1.2. X-ray Diffraction

**Figure 8:**
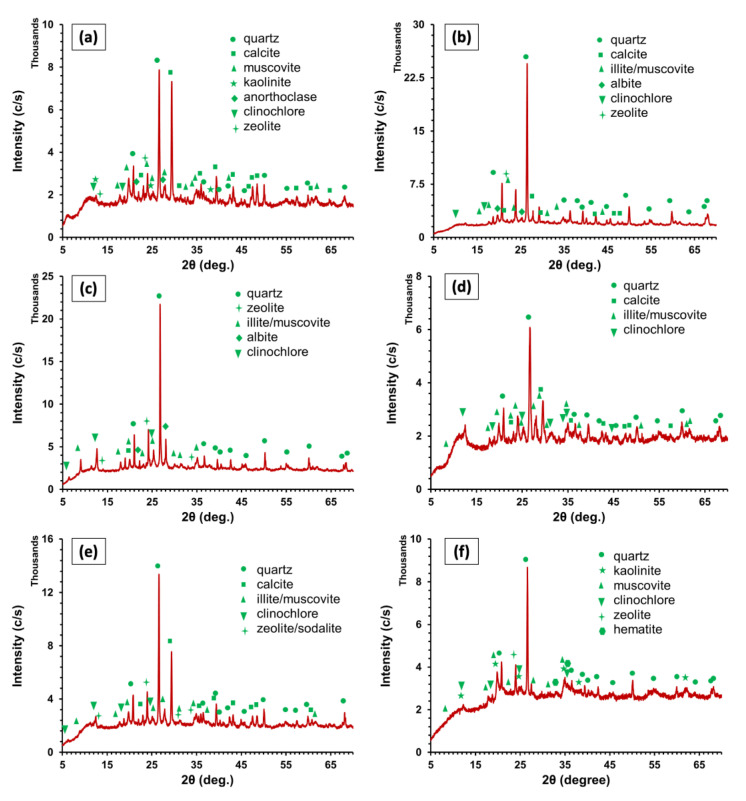
XRD patterns of (**a**): Clay I, (**b**): Clay II, (**c**): Clay III, (**d**): Clay IV, (**e**): Clay V, (**f**): Clay VI.

The XRD patterns of Clay I–VI are displayed in Figure 8a–f, where the quantitative and qualitative phase assessment of multiphase mixtures is carried out. Like XRD patterns of industrial wastes, the ICDD database was used to determine the XRD peaks of the clays used in this study (see Table A1 in Appendix A). Figure 8a–f shows that all clay types have major peaks against quartz, calcite, clinochlore, illite/muscovite, and zeolite, with some minor peaks of albite, anorthoclase, kaolinite, and hematite. Among all clay types, Clay II has the highest amount of crystalline quartz (see Figure 8b), which is also reflected by the highest quantity of SiO_2_ in Clay II. The relatively greater intensity of quartz peak in all clay types can be credited to the presence of SiO_2_. However, the quantity of free SiO_2_ in all clay can be related to the SiO_2_/Al_2_O_3_ ratio and amount of LoI. For instance, Clay II has a higher SiO_2_/Al_2_O_3_ ratio and low LOI due to which Clay II has the highest peak for quartz. The SiO_2_/Al_2_O_3_ ratio of Clay I, Clay III, Clay IV, Clay V, and Clay VI is 2.92, 2.76, 2.73, 3.03, and 1.91, while LoI of mentioned clay types is 22%, 8.9%, 13.19%, 17.3%, and 12.75%, respectively. Based on this, the intensity of quartz in clay specimens can be ordered as Clay III > Clay V > Clay VI > Clay I > Clay IV.

It noteworthy that Clay I has the highest peak against calcite (approximately equal to quartz) (see Figure 8a), but it has a low concentration of CaO as compared to Clay V (see Figure 8e). The proportion of calcite less than 3% may act as a fluxing agent leading to a reduced melting point of clay, while a higher proportion of calcite can increase the porosity of bricks [68]. In addition, higher size (>125 μm) may cause dislocation of CaO from the carbonates, which can hydrate after the firing process leading to variations in the dimensions of the final clay product [68]. This suggests that extra care (such as grinding, pre-processing, and slow firing) should be adopted for preparing brick or other ceramic products with Clay I.

Clay II–V has a mixed layer of illite and muscovite because illite occurs due to the alteration of muscovite in hydrothermal and weathering environments. The presence of illite in an appreciable amount in Clay II–V may produce a higher quantity of glass phase and reduce the melting point. Illite concentration in the mentioned clay types may decrease linear shrinkage attributed to the deformation of pyroplastic. Moreover, illite also reduces the formation of cristobalite and mullite because the presence of a high quantity of SiO_2_ and Al_2_O_3_ has the tendency to form alkaline glass [56]. On the other hand, Clay I/VI only contain muscovite, which reveals the unavailability of hydrothermal and weathering conditions at their respective locations.

Contrary to previous studies on the mineralogy of clays [61,69] where chlorite was the main constituent; in this study, instead of chlorite, clinochlore was found in all clay types. Clinochlore is one of the most abundantly found member of the chlorite group, which is mainly composed of Mg and moderately composed of Si, Al, and Fe. Thus, the peak against clinochlore for the clays studied is in the order of Clay IV > Clay V > Clay I > Clay III > Clay II > Clay VI. The presence of Fe in clinochlore oxidized provides an appealing appearance, which make clays containing clinochlore perfect for decorative and carving stones. Therefore, clinochlore-based clays can be used to prepare bricks for exterior walls (without plaster) for aesthetic purposes.

#### 3.1.3. Thermogravimetric Analysis (TGA)

**Figure 9:**
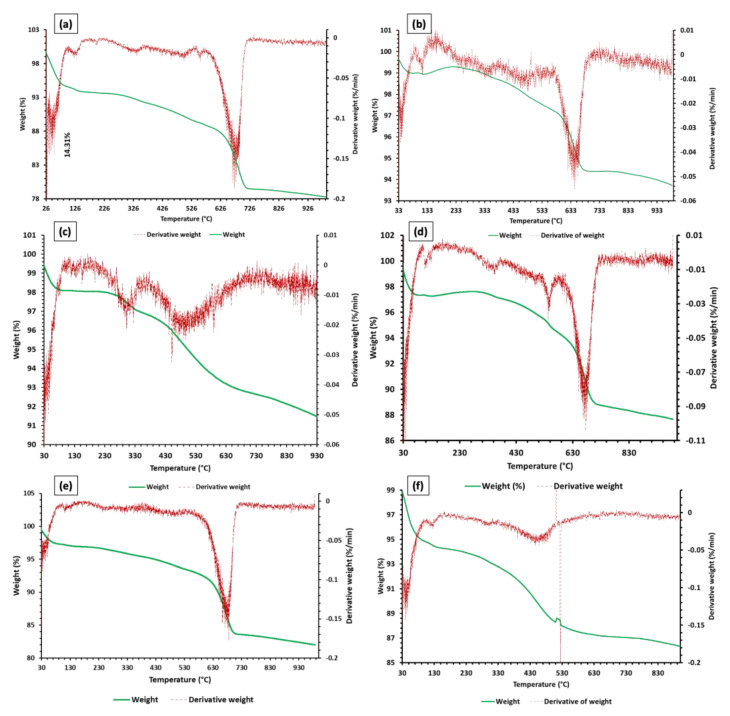
TGA analysis of clay minerals in (**a**): Clay I, (**b**): Clay II, (**c**): Clay III, (**d**): Clay IV, (**e**): Clay V, (**f**): Clay VI.

TGA was used to estimate different types of calcareous and clay minerals. These minerals were distinguished by assessing the temperature at which different peaks appear. Each clay type studied had a different response to temperature and corresponding weight loss (see Figure 9a–f). The total weight loss of Clay I, Clay II, Clay III, Clay IV, Clay V, and Clay VI during TGA is equal to 21.57%, 6.14%, 8.73%, 12.12%, 17.83%, and 13.95%, respectively. The total weight loss is divided into different processes such as oxidation of organic matter, dehydroxylation of the clay minerals, and decomposition of carbonates, hydroxides, sulfides [67,70]. The high weight loss in clay types is greatly influenced by the amount of clay minerals; thus, the order of clay minerals based on weight loss can be ordered as Clay I > Clay V > Clay VI > Clay IV > Clay III > Clay II, which will be discussed in detail in Section 3.1.4.

All clay types experienced a peak due to the expulsion of physically bounded moisture. This peak for Clay I, Clay II, Clay III, Clay IV, Clay V, and Clay VI occurred at 38.5 °C, 40.3 °C, 44.7 °C, 31.6 °C, 39.7 °C, 41.1 °C with corresponding weight loss of 3.3%, 0.63%, 1.4%, 1.58%, 0.94%, and 2.94%, respectively. This shows that the hydroscopic nature of Clay I/VI, Clay III/IV, and Clay II/V is high, moderate, and low, respectively. However, weight loss of clays due to the expulsion of water can also be related to the presence of moisture available nearby the respective clay location [71]. It is important to indicate that the temperature and corresponding weight loss of clays studied is appreciably lower than previously studied clays in Turkey [69], wherein the peak was observed at 111.3 °C with 7% weight loss.

The second lower intensity was observed in Clay I, Clay II, Clay IV, and Clay VI, but negligible in Clay III and Clay V. This peak occurred due to the decomposition of Al and Fe hydroxides at a temperature of 130.1 °C, 116.21 °C, 136.4 °C, 114.1 °C, 112.2 °C, 125.7 °C for Clay I, Clay II, Clay III, Clay IV, Clay V, and Clay VI, respectively. The associated weight loss of Clay I, Clay II, Clay III, Clay IV, Clay V, and Clay VI due to the decomposition of Al and Fe hydroxides was 0.4%, 0.08%, 0.04%, 0.05%, 0.18%, and 0.27%, respectively [72]. It is noteworthy that the presence of Al and Fe hydroxides is disadvantageous because they require excessive energy for decomposition. Additionally, the formation of corresponding oxides such as Al_2_O_3_ and Fe_2_O_3_ increases refractoriness and LoI of fired clay products [64].

Small peaks for all clay types (except Clay III) can be seen between temperature ranges of 200–300 °C and 300–550 °C, which is due to the combustion of organic content and discharge of chemically bound water [73]. The total weight loss due to the mentioned phenomenon and temperature range (200–550 °C) for Clay I, Clay II, Clay IV, Clay V, and Clay VI is 4.38%, 1.72%, 1.59%, 2.93%, and 5.01%, respectively. Clay types (Clay I, Clay V, and Clay VI) have higher weight loss due to the high LoI values. Based on the LoI values, the order of weight loss for the three mentioned clay types should be Clay I > Clay V > Clay IV, but based on the weight loss values, the order is Clay VI > Clay I > Clay V. This can be ascribed to a greater amount of chemically bonded water in Clay IV as compared to Clay VI. In Clay III, the weight loss due to the combustion of organic content and expulsion of chemically bonded water occurred at temperature ranges of 200–375 °C and 400–500 °C with a corresponding weight loss of 1.24%.

An abrupt and highest weight loss for Clay I, Clay II, Clay III, Clay IV, Clay V, and Clay VI occurred at 684.67 °C, 646.46 °C, 451.71 °C, 675.81 °C, 685.68 °C, and 524.96 °C with a corresponding weight loss of 8.65%, 2.77%, 1.98%, 4.95%, 8.55%, and 1.02%, respectively. The weight loss at the mentioned temperature for each clay type is due to the dehydroxylation of illite and clinochlore [74].

#### 3.1.4. Particle Size Distribution

**Figure 10:**
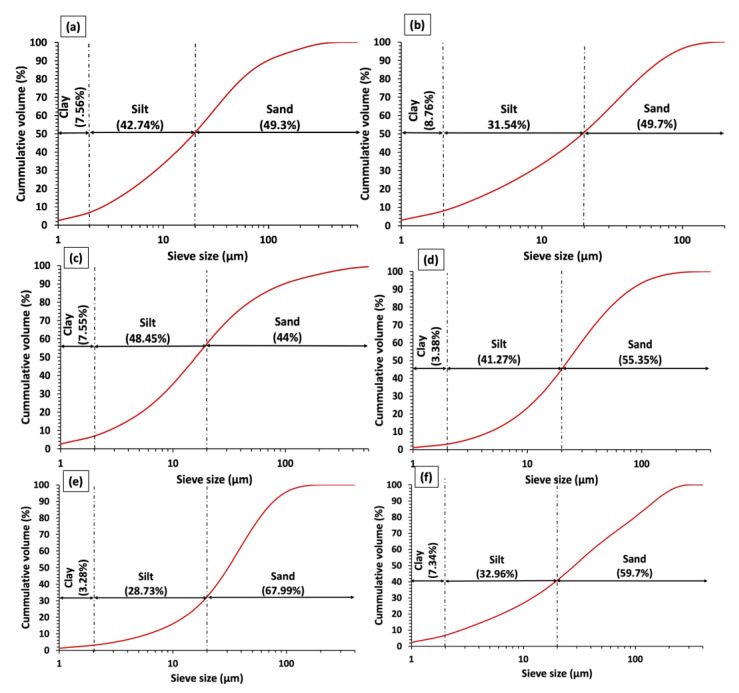
Particle size distribution of (**a**): Clay I, (**b**): Clay II, (**c**): Clay III, (**d**): Clay IV, (**e**): Clay V, (**f**): Clay VI.

Figure 10a–f presents the gradation curve of clay specimens, wherein variable sand, silt, and clay fractions are specified as per the International Society of Soil Science [75]. The plotted gradation curves are required to critically examine the different particle sizes in a clay specimens and evaluate their effect on the parameters that impact properties such as swell behavior, activity, and PI. In view of this, the mean sizes of sand, silt, and clay fractions contained in the specimens and the mean particle size of each specimen were considered as a whole. According to Figure 10a–f, the sand fraction of clay specimens is in the order of Clay V > Clay VI > Clay IV > Clay II > Clay I > Clay III, which are related to the particles with an equivalent spherical diameter greater than 20 μm. The silt fraction is in the order of Clay III > Clay I > Clay IV > Clay VI > Clay II > Clay V, which represents the particles with a diameter between 2 μm and 20 μm. Finally, the clay fraction is in the order of Clay II > Clay I > Clay III > Clay VI > Clay VI > Clay V, which is characterized by the particle size less than 2 μm. It noteworthy that clay fractions between 16% to 40% is considered favorable for the clay-based materials [76], which is not the case in clays studied.

The clay fractions are very fine clay materials and are highly dependent on the plasticity of clayey materials. For instance, Clay II should have the highest plasticity, which is not the case as per Section 3.2. This suggests that along with clay fraction, silt fraction is also an important factor influencing the plastic behavior of clayey soils. Moreover, an appreciable amount of silt fraction in clay has a positive influence on the glass formation, body skeleton of clay, and reduction in vitrification temperature, provided that such clay fractions contain an appreciable amount of quartz [77]. The total clay and silt fractions in Clay I, Clay II, Clay III, Clay VI, Clay V, and Clay VI are 50.28%, 40.3%, 56%, 44.65%, 32.01%, 40.3%; therefore, as per the particle size distribution of clay specimens, the plasticity of clays can be ordered as Clay III > Clay I > Clay VI > Clay II/VI > Clay V.

**Figure 11:**
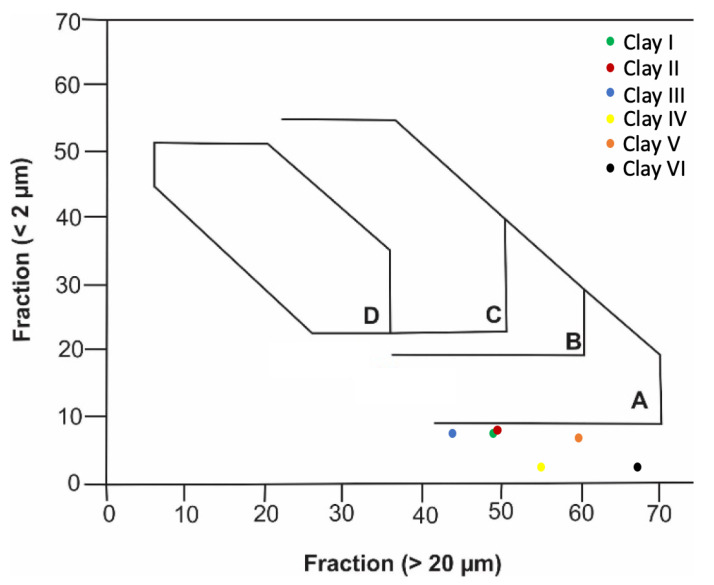
Suitability of clays studied for different ceramic products according to Winkler’s diagram.

The particle size distribution of clays is also essential for determining their suitability for different ceramic applications because it could indicate the firing and drying behavior of clays and the mechanical properties of clay-based products [76]. The particle sizes for clay specimens were plotted in Winkler’s diagram to determine their suitability for different clay products (see Figure 11) such as solid bricks (A), vertically performed bricks (B), roofing tiles and lightweight blocks (C), and thin-walled hollow bricks (D). According to Figure 11, Clay I, Clay II, Clay III, and Clay V can nearly be used for solid bricks. On the other hand, Clay IV and Clay VI cannot be used for the mentioned clay-based products, which can be attributed to the presence of a low percentage of clay fraction. To make studied clays suitable for the ceramic industry, different percentages of materials (depending on the type of application) with particle sizes less than 2 μm should be added, as shown in Figure 11. In other words, the clay types studied require modification and treatment to produce clay-based products for non-structural and structural applications.

### 3.2. Index Properties

Index properties of clays play an important role in determining the manufacturing of clay-based products [78]. In addition, the index properties are critical to evaluate the drying behavior, workability, and ceramic application of clays [79]. The index properties of clays include LL, PL, PI, and SL. The factors that influence the index properties of clays include mineral composition, particle size distribution, organic matter, origin of geological formation, and impurities [80].

**Figure 12:**
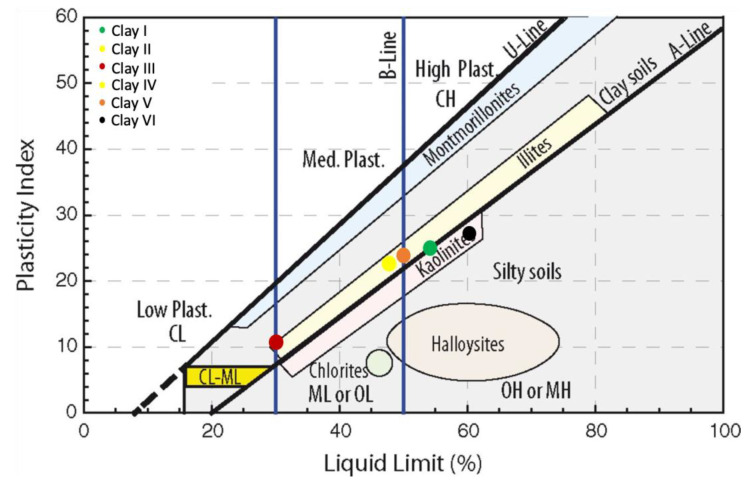
Location of clay studied on combined Holtz and Kovacs diagram and Casagrande plasticity chart.

The values for LL, PL, PI, and SL are listed in Table A2 (see Appendix A). Table A2 shows that the LL, PL, and PI are highest for Clay VI while the shrinkage limit is highest for Clay V. On the other hand, the LL, PL, and PI are lowest for Clay III and SL is lowest for Clay VI. The combination of Holtz and Kovacs diagram and Casagrande plasticity chart was used to plot the intersection of LL and PI to determine the plasticity extent of clay specimens, as shown in Figure 12. Figure 12 shows that Clay I, Clay V, and Clay VI are high plastic clays while Clay II, Clay II, and Clay IV are medium plastic clays.

A high LL (>50%) usually suggests high swelling/shrinkage potential and high compressibility, which indicates that Clay I, Clay V, and Clay VI cannot be used in the ceramic industry without excessive compaction and implementation of improvement strategies to eliminate the risk of swelling or shrinkage. Moreover, clays with high PI generally result in low shear strength, which means that a small change in moisture percentage can considerably change the consistency [41]. Therefore, Clay I, Clay V, and Clay VI cannot be placed under the foundation of structures. It is important to note that all clay specimens also have a PL greater than 20%, which is important for ceramic applications because it determines the minimum amount of moisture required to achieve plastic conditions and high PL clays are quite difficult to dry [69]. Contrary to this, high plastic clays decrease the wearing of equipment required for milling and conformation (extruder) and possess high mechanical strength [60]. In the case of SL, the clay studied followed a similar pattern as per previous studies [81], wherein clays with higher PI have lower SL see Table A2. This is because clays with high PI would be in a plastic state at a wide range of moisture content, which varies from LL to PL; thus, require very little change in moisture content to transform into a solid state (i.e., low SL).

**Figure 13:**
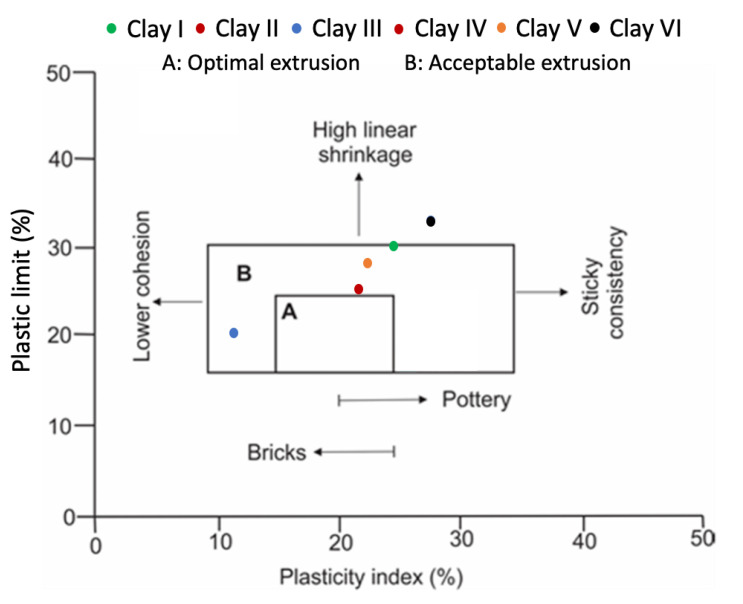
Extrusion prognostic (or workability chart) of clays studied via index properties.

Figure 13 shows an extrusion prognostic (or workability chart) of clay specimens using index properties (PL and PI). PL is associated with the quantity of moisture needed for clay to achieve the consistency that facilitates extrusion process. While the PI corresponds to the range between plastic and sludge consistency. Therefore, for practical reasons, PI must be higher than 10% for the clays to be used in the ceramic industry [82] because extremely low PI can make the extrusion process difficult. These challenges are associated with a possible change in the quantity of extrusion water, which may cause unfavorable dimensional properties and even propagate deterioration in the green pieces. In this study, all clay types have PI higher than 10%, as shown in Table A2 and Figure 13, which will improve the efficiency of the extrusion process during the production of clay-based products. Figure 13 shows that only Clay IV is close to the optimal extrusion region while Clay I, Clay II, Clay III, Clay VI, Clay V are located in the acceptable extrusion region. On the other hand, Clay IV are unsuitable for extrusion owing to their high plasticity parameters making it unfit for the ceramic industry. Moreover, Figure 13 also shows that all clay types (except Clay III and Clay VI) are good for both pottery and brick production, while Clay III is maybe only acceptable for brick production.

To decrease the plasticity parameters of clay specimens and increase their chances of usability in the ceramic industry and brick production, FA, FS, and SF were substituted in a percentage between 10–50% with an increment of 10%. Table A2 shows the influence of the mentioned waste materials on the index properties of clay specimens. As per Table A2, SF tends to negligibly decrease the plasticity of clays at 10% incorporation level, and a further increase in SF leads to an excessive increase in plasticity and SL. The decrease in the index properties of clay specimens containing 10% SF is due to the substitution of non-expansive SF particles. In addition, the incorporation of SF coats clay particles having large particle sizes with appreciable cementitious value, which is known as a pozzolanic reaction between aluminous minerals in clays and SF [83]. For example, incorporation of 50% SF increases the LL, PL, PI, and SL of Clay I by 23.63%, 33.33%, 12.1%, and 62.5%, respectively, as compared to pure clay, and similar behavior was observed in other clay specimens. The maximum increase in index properties with the addition of SF was observed in Clay III, wherein incorporation of 50% SF increased LL, PL, PI, and SL of Clay III by 58.06%, 75.01%, 27.27%, and 80%, respectively as compared to the pure clay specimen. This suggests that addition of SF does not improve the properties of clay specimen to be used in the ceramic industry. It is noteworthy that the effect of adding SF on the index properties contradicts previously published studies on similar topic [84], which is due to the different types and relative amount of silicate clay minerals in the clays studied. It is interesting to indicate that incorporation of 30% SF is most favorable for all types of clays because it has a negligible impact on increasing the index properties.

On the other hand, incorporation of FS and FA proved to be favorable in reducing the plasticity of clay specimens. For example, incorporation of 50% FA decreased the LL, PL, PI, and SL of Clay I by 32.72%, 33.33%, 32%, and −15%, respectively, as compared to the pure clay specimen, which is similar to the results reported by Phanikumar and Nagaraju [52]. Similarly, the incorporation of 50% FS decreased LL, PL, PI, and SL of Clay I by 38.18%, 43.33%, 32.11%, and 5.11%, respectively, as compared to the pure clay specimen. The decrease in index properties due to the incorporation of FA and FS can be attributed to their pozzolanic nature that induces flocculation leading to an increase in the size of the blended particle.

The addition of FA led to fluctuations in index properties, particularly for PL and SL for Clay I, Clay III, Clay IV, Clay V, and Clay VI, which can be attributed to the complex mineralogy of the mentioned clay types. Moreover, the decrease in index properties clays is appreciable with 20% FA and a further increase in FA has a negligible impact. While FS-modified clays showed a consistent reduction trend in index properties without any fluctuations. Moreover, in the case of FS-incorporated clays, the decrease in index properties is gradual rather than rapid as in the case of FA- and SF-modified clays.

**Figure 14:**
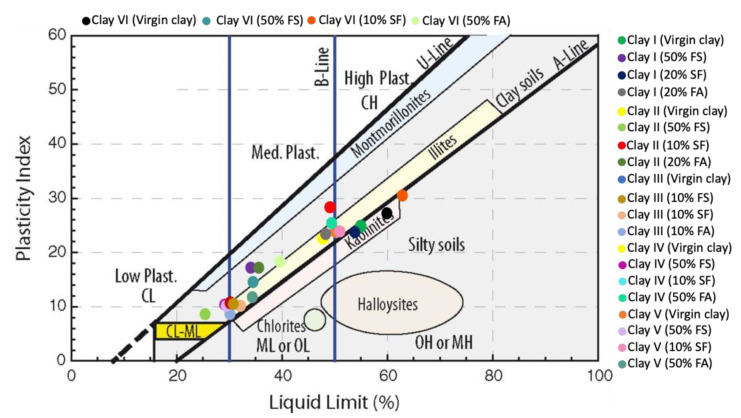
Location of clay and modified clay specimens on the combined Holtz and Kovacs diagram and Casagrande plasticity chart.

In order to see the impact of optimal percentage incorporation of waste materials on the plasticity and transformation between clays, the location of waste material-modified clays was plotted on combined Holtz and Kovacs diagram and Casagrande plasticity chart using PI and LL (see Figure 14). Figure 14 shows that incorporation of FA and FS changes clay type from inorganic clay with high plasticity to inorganic clay with medium and low plasticity, while the incorporation of SF results in opposite except for Clay I. This is because of the substitution of fine-grained particles of clays with coarse-grained particles of FA and FS, which leads to low expansive clays from medium expansive clays. In comparison to FA, FS is more effective in achieving the mentioned attributes (see Figure 14).

The incorporation of FA, SF, and FS in clays may improve their extrusion prognostic. For example, incorporation of 20–30%, 10–20%, 10–30%, 10–30%, and 30–50% FS may locate Clay I, Clay II, Clay IV, Clay V, and Clay VI inside optimal extrusion of workability chart. Incorporation of 30–50%, 10–20%, 20–40%, 10–30%, 30–50% FA in Clay I, Clay II, Clay III, Clay IV, Clay V, Clay VI will place them in optimal extrusion region of workability chart. Incorporation of other FS and FA percentages in different clay specimens can also place them in acceptable extrusion region of workability chart. However, 10% SF modification can place Clay IV in the optimal extrusion region of the workability chart.

## 4. Conclusions

The mineralogy and index properties of five unexplored clay samples from different locations in Turkey and sixth clay specimen from Siberia were investigated. In addition, the efficiency of FA, FS, and SF in improving the index properties of clays were also studied. The following conclusions can be deduced from the above discussion:

1. Clay I, Clay IV, and Clay V have a lower amount of Al_2_O_3_ and considerable alkaline earth oxides (CaO and MgO) and alkali oxides (Na_2_O and K_2_O), which lead to the formation of the vitreous phase and reduce the melting point of clays required for the production of bricks.
2. All clay types studied have quartz, clinochlore, and illite/muscovite as mutual accessory minerals. Some clay types have variable minerals such as Clay I/VI has kaolinite, Clay I/II/III/VI has zeolite, Clay III has albite, and Clay VI has hematite. However, the mentioned clay minerals are in negligible amounts, which will not appreciably impact the properties of clay-based products.
3. Clay II, Clay IV, and Clay V are medium plastic clays while Clay I, Clay III, and Clay VI are high plastic clays, which are fine to be used in medium quality and low-quality clay-based products, respectively. However, the incorporation of 10–30% FS/FA in clay specimens reduces the index properties, which can be used to produce high-quality clay products. On the other hand, SF does not have a positive impact on the index properties of clays except Clay I.
4. The addition of 10–30% FA/FS can also improve the workability of clays by placing them in the optimal extrusion region of the workability chart, which is beneficial in processing clays for different clay-based products.

## Figures and Tables

**Table 1:** Physical properties of clay specimens.

Property	Designation of Clay Specimens					
	Clay I	Clay II	Clay III	Clay IV	Clay V	Clay VI
Location	Bartin University	Üçyildiz	Işıklar	Boyabat	Boyabat	Sjenica Serbia
Gravel size (%)	0	0	0	0	0	0
Sand size (4.75–0.075 mm) (%)	49.3	49.7	44	55.35	67.99	59.7
Silt size (0.075–0.005 mm) (%)	42.74	31.54	48.45	41.27	28.73	32.96
Clay size (<0.005 mm) (%)	7.56	8.76	7.55	3.38	3.28	7.34
USCS classification	MH	CL	CL	CL	CL	MH
Specific gravity	2.663	2.685	2.706	2.793	2.750	2.716
Moisture content (%)	6.1	2.0	2.5	3.5	4.3	6.9
Surface area (m^2^/g)	29.563	23.03	20.39	22.73	30.49	64.49

**Table 2:** Physical properties of FA, FS, and SF.

Property	Material		
	FA	FS	SF
Specific gravity	2.026	3.327	2.172
Moisture content (%)	0.3	0.1	0.3
Surface area (m^2^/g)	0.18	0.55	0.13

## Data Availability

Data will be made available on request.

## Appendix A

**Table A1:** ICDD reference for the peaks in XRD patterns of raw materials.

Sr. No.	Raw Materials	Phases (ICDD Reference)
1	Clay I	Quartz (01-078-1252) Calcite (01-086-2334) Muscovite (01-084-1302) Kaolinite (00-029-1488) Anorthoclase (01-075-1634) Clinochlore (00-045-1321) Zeolite (00-045-0510) Sodalite (00-052-0145) Albite (00-001-0739) Illite (00-043-0685) Hematite (00-039-0238)
2	Clay II	
3	Clay III	
4	Clay IV	
5	Clay V	
6	Clay VI	
1	SF	Amorphous structure
2	FC	Forsterite (01-083-1535) Spinel (01-075-1798) Amorphous structure
3	FA	Quartz (01-089-8936) Mullite (01-079-1275) Zeolite (00-035-0060) Chlorite (00-042-0584) Amorphous structure

**Table A2:** Index properties of FS, SF, and FA modified clays.

Clays	Waste Materials	Percentage Replacement (%)	LL	PL	PI = LL − PL	SL
CLAY I	FS	0	55	30	25	20
		10	52	25	26	22
		20	46	21	25	21
		30	45	20	24	21
		40	38	18	20	21
		50	34	17	17	19
	SF	0	55	30	25	20
		10	55	29	26	24
		20	54	31	23	26
		30	58	34	24	31
		40	61	37	24	34
		50	68	40	28	39
	FA	0	55	30	25	20
		10	53	25	28	24
		20	48	26	23	23
		30	47	23	24	23
		40	42	21	21	23
		50	37	20	17	23
CLAY II	FS	0	48	26	22	23
		10	37	19	18	19
		20	34	18	15	18
		30	30	17	13	18
		40	28	16	11	19
		50	25	16	9	17
	SF	0	48	26	22	23
		10	49	21	28	21
		20	53	26	27	25
		30	57	29	28	30
		40	60	32	28	31
		50	68	36	32	40
	FA	0	48	26	22	23
		10	38	19	18	19
		20	36	20	17	20
		30	34	19	14	20
		40	33	18	14	20
		50	28	18	10	20
CLAY III	FS	0	31	20	11	20
		10	29	20	9	20
		20	28	19	9	19
		30	25	18	7	19
		40	23	17	6	18
		50	21	16	5	18
	SF	0	31	20	11	20
		10	32	22	10	21
		20	38	25	13	25
		30	38	26	11	27
		40	45	31	14	33
		50	49	35	14	36
	FA	0	31	20	11	20
		10	30	21	9	22
		20	30	22	8	19
		30	27	21	6	23
		40	26	20	6	23
		50	25	21	5	23
CLAY IV	FS	0	48	26	22	23
		10	43	23	20	26
		20	40	22	19	23
		30	37	20	16	23
		40	33	21	11	23
		50	29	18	11	21
	SF	0	48	26	22	23
		10	49	25	24	25
		20	53	27	25	26
		30	54	28	26	34
		40	61	33	28	35
		50	62	34	28	41
	FA	0	48	26	22	23
		10	46	26	20	26
		20	43	24	19	25
		30	40	24	16	26
		40	38	22	16	25
		50	35	22	13	26
CLAY V	FS	0	50	27	23	24
		10	44	24	20	23
		20	41	24	17	22
		30	37	20	17	22
		40	33	20	13	20
		50	29	19	10	19
	SF	0	50	27	23	24
		10	51	27	23	24
		20	54	29	25	29
		30	57	36	22	34
		40	61	37	25	37
		50	66	43	23	42
	FA	0	50	27	23	24
		10	46	24	22	24
		20	43	23	20	22
		30	42	22	19	24
		40	37	23	14	23
		50	34	22	12	23
Clay VI	FS	0	60	33	27	17
		10	58	28	30	19
		20	50	26	24	18
		30	44	22	21	18
		40	40	20	21	18
		50	34	18	15	19
	SF	0	60	33	27	17
		10	63	32	31	23
		20	65	32	33	41
		30	66	35	31	34
		40	71	37	34	38
		50	71	37	34	40
	FA	0	60	33	27	17
		10	58	28	30	19
		20	52	27	23	19
		30	48	24	25	18
		40	44	21	23	19
		50	39	21	18	19
